# Efficacy of the Modified 5-Item Frailty Index in Predicting Surgical-Site Infections in Patients Undergoing Breast Implant Augmentation: A National Surgical Quality Improvement Project-Based 5-Year Study

**DOI:** 10.1093/asjof/ojad067

**Published:** 2023-07-18

**Authors:** Helen Liu, Arya Akhavan, Raymond Yin, Taylor Ibelli, Max Mandelbaum, Abigail Katz, Suhas Etigunta, Eric Alerte, Annet Kuruvilla, Chuanju Liu, Peter J Taub

## Abstract

**Background:**

The ability to predict breast implant augmentation complications can significantly inform patient management. A frailty measure, such as the modified 5-item frailty index (mFI-5), is becoming an increasingly established risk factor for adverse postoperative outcomes. The authors hypothesized that the mFI-5 is predictive of 30-day postoperative complications in breast augmentation.

**Objectives:**

To investigate if mFI-5 can predict the likelihood and magnitude of 30-day complications resulting from breast augmentations.

**Methods:**

A retrospective review study of the National Surgical Quality Improvement Program database for patients who underwent breast implant augmentation without other concurrent procedures, from 2015 to 2019. Age, BMI, number of major comorbidities, American Society of Anesthesiologists (ASA) classifications, smoking status, mFI-5 score, and modified Charlson comorbidity index score were compared as predictors of all-cause 30-day complications and 30-day surgical-site complications using regression analyses.

**Results:**

Overall, 2478 patients were analyzed, and among them, 53 patients developed complications (2.14%). mFI-5 score significantly predicted surgical-site infection (SSI) complications (odds ratio [OR] = 4.24, *P* = .026). Frail patients had a higher occurrence of SSIs than nonfrail patients (*P* = .049). Multivariable analyses showed ASA class predicted 30-day SSI complications (OR = 5.77, *P* = .027) and mFI-5 approached, but did not reach full significance in predicting overall 30-day complications (OR = 3.14, *P* = .085).

**Conclusions:**

To date, the impact of frailty on breast implant procedure outcomes has not been studied. Our analysis demonstrates that the mFI-5 is a significant predictor for SSIs in breast implant augmentation surgery and is associated with overall complications. By preoperatively identifying frail patients, the surgical team can better account for postoperative support to minimize the risk of complications.

**Level of Evidence: 4:**

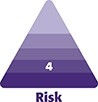

Breast augmentation is one of the most frequently performed elective plastic surgery procedures in the United States. In 2021, over 350,000 breast augmentations were performed, producing approximately 1.5 billion dollars in revenue.^1^ Despite this, there are very few multicenter national studies investigating risk factors for breast augmentation complications.

Breast augmentations differ in the types of implants used (shape, filler, shell, anterior-posterior profile), incisions and breast pocket placement, antibiotic regime, and postoperative instructions.^2,3^ Implant rupture rates range from 1.1% to 17.7% after primary augmentation and 2.9% to 14.7% after revision augmentation.^4^ Studies show that capsular contracture has incidence rates ranging from 0% to 20%, some noting almost 50% complications by 10 years.^5-7^ Reoperation rates for primary breast augmentations approach 20% and are higher for subsequent augmentations.^8,9^ Other common complications include infection, rippling, seroma, hematoma, nerve injury, and implant malposition.^2,3^ Thus, breast augmentation procedures have the highest complication rate of all aesthetic procedures, emphasizing the importance of understanding the factors that contribute to adverse outcomes, and thus ensuring better patient safety.

Frailty is increasingly recognized as an important risk factor for adverse events.^10^ Frailty has been shown to be a risk factor for complications across multiple surgical specialities,^11-17^ and for historically low-risk or elective procedures.^18-20^ Many frailty measurement tools have been developed for use in research studies, including the frailty index scoring system (modified 5-item frailty index, mFI-5) developed by Velanovich et al using the American College of Surgeons’ (ACS) National Surgical Quality Improvement Program (NSQIP) database.^21^

To our knowledge, the impact of frailty on breast augmentation has not been studied. Using the NSQIP database, we sought to understand the association between frailty and 30-day complications after breast augmentation procedures. The authors hypothesized that mFI-5 significantly predicts 30-day complications after breast augmentation procedures. We also sought to compare other risk indices such as smoking, age, BMI, diabetes, steroid use, American Society of Anesthesiologists (ASA) class, etc, to better assess the predictive value of the mFI-5 in breast augmentations.

## METHODS

### Study Design and Data Source

Approval was obtained from the Institutional Review Boards of the Icahn School of Medicine (No. 21-01655, Mount Sinai Hospital, New York, NY). This study was exempt from requirements for consent, as the data from ACS NSQIP are deidentified and compliant with the Health Insurance Portability and Accountability Act.

### Data Extraction

NSQIP files from 2015 to 2019 were extracted (AC S, Chicago, IL). This database includes detailed information regarding surgical outcomes, demographic data, and perioperative and postoperative patient information. Years 2015 to 2019 were used because the data were standardized in 2015 and contained the same preoperative and postoperative data points. Services and diagnoses were determined through Current Procedural Terminology (CPT) codes, International Classification of Diseases, Ninth and Tenth Revision, Clinical Modification codes (ICD-9-CM, US health system’s adaptation of the international ICD-9 standard and ICD-10-CM, US health system’s adaptation of the international ICD-10 standard), and standardized billing items.

Study inclusion criteria included patient records with the CPT procedure code for elective breast implant augmentation (CPT code 19325) from 2015 to 2019. All other codes were excluded from this population-based retrospective cohort study. Records with ICD-9 or ICD-10 codes for breast reconstruction diagnoses were excluded (174.0, 174.9, 610.0, 610.8, 612.0, 612.1, V10.3, V45.71, V50.41, V51.0). Males and patients without sex information were also excluded.

#### Study Variables

Variables such as age, gender, smoking status, height, weight, race, ethnicity, and ASA classification were collected. Age was separated into 4 categories: <30 years old, 30 to 49 years old, 50 to 65 years old, and >65 years old. BMI was calculated based on the provided height and weight data. Patients were aggregated into 3 categories: healthy weight or underweight (<24.9), overweight (25.0-29.9), and obese (≥30.0). Patients were scored on a scale from 0 to 5 based on the number of mFI-5 factors present: History of congestive heart failure (CHF), hypertension (HTN), chronic obstructive pulmonary disease (COPD), diabetes, and dependent functional status. Patients were then separated into 2 categories: 0 and ≥1 factors. The number of comorbidities was sorted into 3 categories: 0, 1, and ≥2 major comorbidities. Patients were also given a modified Charlson comorbidity index (mCCI) score based on their age, history of CHF, COPD, chronic kidney disease, liver disease, diabetes, and metastatic cancer status. Patients were separated into 3 categories: 0, 1, and ≥2. ASA class was separated into 3 categories: Class I, Class II, and Class III or greater.

#### Outcomes

Overall 30-day complication outcomes were categorized based on the presence or absence of any negative outcome recorded in the NSQIP database. Surgical-site infection (SSI) outcomes were also categorized on presence or absence of wound dehiscence or wound infection complications. Clavien–Dindo scores, a measure of severity of complications, were calculated based on patient postoperative outcomes. Furthermore, we looked into risk predictors and their effect on procedure logistics such as readmission, reoperation, and overnight stay (length of hospital stay >0). Study objectives and design, including population, intervention, and outcomes of interest, were established before data access.

### Statistical Analysis

All analyses were performed using R version 4.0.3 (R Core Team 2021). Continuous variables were reported as mean ± standard deviation and median. Categorical variables were reported as frequencies and percentages. Univariable associations between study variables were analyzed using Mann–Whitney *U* tests for continuous variables and Fisher's exact tests for categorical variables, respectively. Univariable logistic regression analysis was used to quantify risk factors for outcomes by each stratum. Multivariable logistic regression analysis was conducted to calculate the odds ratios (ORs) and 95% CIs when controlling for confounding variables. Variables included in the multivariate regression analysis included: mFI-5, age, obesity (BMI ≥ 30), smoking. Statistical significance was set at *P* < .10.

## RESULTS

A total of 2478 patients were identified. The age range was 18 to 78, and mean age was 35.94 ± 11.34 years (Table 1). The mean BMI was 23.39 ± 4.45 kg/m^2^. Most patients identified as White (*n* = 1780, 71.83%) and non-Hispanic (*n* = 1744, 70.38%). Descriptive statistics for the study population are summarized in Table 1.

**Table 1.** Patient Characteristics

Variable	Breast implant augmentation (*n* = 2478)	
Patient demographics	*n*	%
Age (years)^a^	35.94 ± 11.34	
Race American Indian or Alaska Native Asian Black or African American Native Hawaiian or Pacific Islander White Unknown/not reported	3 96 135 2 1780 462	0.12 3.87 5.45 0.08 71.83 18.64
Ethnicity Hispanic Non-Hispanic Unknown	160 1744 574	6.46 70.38 23.16
BMI (kg/m^2^)^a^	23.39 ± 4.45	
Height (inches)^a^	63.29 ± 14.57	
Weight (lbs)^a^	137.75 ± 33.02	
Smoking status Smoker Nonsmoker	301 2177	12.15 87.85

SD, standard deviation. ^a^Reported mean ± SD.

### Distribution of Surgical-Risk Indices

Most patients were nonfrail (*n* = 2338, 94.35%), followed by an mFI-5 score of 1 (*n* = 128, 5.17%), and mFI-5 = 2 (*n* = 12, 0.48%; Table 2). Similarly, most patients had an mCCI score of 0 (*n* = 2080, 83.94%), followed by mCCI = 1 (*n* = 250, 10.09%), mCCI = 2 (*n* = 98, 3.95%), and greater than or equal to mCCI = 3 (*n* = 50, 2.02%). 2319 patients (93.58%) had no comorbidities, 138 (5.57%) had 1 comorbidity, 22 (0.89%) had 2 or more comorbidities. Among NSQIP variables collected, hypertension, diabetes, and steroid use were the most common comorbidities (4.52%, 1.33%, and 0.69%, respectively). 1301 (52.50%) patients were categorized under ASA classification of no disturbance, 1055 (42.57%) under mild disturbance, 113 (4.56%) under severe disturbance, and 3 patients (0.12%) with an ASA classification = IV. 301 patients (12.15%) identified as current smokers within 1 year of their procedure.

**Table 2.** Surgical-Risk Indices

Risk strata	Stratification	*n*	%
mFI-5 score	0	2338	94.35
	1	128	5.17
	2	12	0.48
mCCI score	0	2080	83.94
	1	250	10.09
	2	98	3.95
	≥3	50	2.02
No. of major comorbidities	0	2319	93.58
	1	138	5.57
	≥2	22	0.89
ASA class	I	1301	52.50
	II	1055	42.57
	III	113	4.56
	IV	3	0.12
Smoking	Nonsmoker	2177	87.85
	Smoker	301	12.15
Comorbidities	Hypertension	112	4.52
	Diabetes	33	1.33
	Steroid use	17	0.69
	Dyspnea	9	0.36
	Chronic obstructive pulmonary disease	6	0.24
	Bleeding disorder	4	0.16
	Disseminated cancer	2	0.08
	Dependent functional status	1	0.04

ASA, American Society of Anesthesiologists; mCCI, modified Charlson comorbidity index; mFI-5, modified 5-item frailty index.

We found an overall 30-day aggregate complication rate of 2.14%. The most common individual complications were unplanned return to the OR (*n* = 27, 1.09%), readmission within 30 days (*n* = 14, 0.56%), and superficial SSI (*n* = 8, 0.32%; Table 3). We see a higher percentage of surgical-site complications in the frail group when compared with the nonfrail group (*P* = .049; Table 4).

**Table 3.** Study Cohort Outcomes

Outcomes	Overall cohort	
	*n*	%
DVT	1	0.04
Bleed	1	0.04
Superficial SSI	8	0.32
Deep SSI	2	0.08
Organ space SSI	1	0.04
Sepsis	1	0.04
*Clostridium difficile* infection	1	0.04
UTI	6	0.24
Wound dehiscence	4	0.16
Readmission	14	0.56
Unplanned return to OR	27	1.09

DVT, deep vein thrombosis; OR, operating room; SSI, surgical-site infection; UTI, urinary tract infection.

**Table 4.** Outcomes by Frailty Score

Outcomes	Overall cohort (*n* = 2478)		mFI = 0 (*n* = 2338)		mFI ≥ 1 (*n* = 140)		*P*-value
	*n*	%	*n*	%	*n*	%	
Overall 30-day complication	53	2.14	47	2.01	6	4.29	.120
Surgical-site complication	15	0.61	12	0.51	3	2.14	.049^a^
Mild systemic complication	10	0.40	9	0.38	1	0.71	.442
Clavien–Dindo ≥3 complication	40	1.61	37	1.58	3	2.14	.492

mFI, modified frailty index. ^a^Fisher exact test comparing frailty groups.

### Univariate Logistic Regression Analysis of Surgical-Risk Indices Outcomes

To determine the associated risk of 30-day complications within each stratum, univariate logistic regression analysis was performed. We used age <30 years, BMI < 25 kg/m^2^, frailty score of 0, mCCI score of 0, no comorbidities, nonsmoking status, and ASA Class I as reference groups (Table 4). Age was determined to be the best predictor of overall 30-day complications (age > 65, OR = 5.86, 95% CI: 1.27-20.42, *P* = .010). Other variables did not reach significance (Table 5).

**Table 5.** Univariate Logistic Regression Analyses for Overall Complications by Strata

Risk index	Stratification	*n*	All complications	
			Odds ratio (95% CI)	*P*-value
Age, years	<30 (Ref)	1326	N/A	N/A
	30-49	818	2.51 (1.26-5.57)	.014
	50-65	285	1.61 (0.49-4.68)	.400
	>65	49	5.86 (1.27-20.42)	.010
BMI, kg/m^2^	<24.9 (Ref)	1819	NA	NA
	25.0-29.9	446	1.45 (0.73-2.67)	.259
	>30	192	0.76 (0.18-2.14)	.657
No. of major comorbidities	0 (Ref)	2319	NA	NA
	1	138	2.25 (0.85-4.97)	.068
	≥2	21	2.47 (0.14-12.25)	.382
Smoking status	Nonsmoker (Ref)	2177	NA	NA
	Smoker	301	1.49 (0.68-2.95)	.279
ASA class	I—no disturb (Ref)	1301	NA	NA
	II—mild disturb	1055	1.34 (0.77-2.37)	.301
	≥III—severe disturb	116	1.34 (0.32-3.91)	.636
mFI-5 score	0 (Ref)	2338	NA	NA
	≥1	140	2.18 (0.82-4.82)	.078
mCCI score	0 (Ref)	2080	NA	NA
	1	250	0.97 (0.33-2.24)	.944
	≥2	148	1.66 (0.57-3.87)	.294

ASA, American Society of Anesthesiologists; mCCI, modified Charlson comorbidity index; mFI-5, modified 5-item frailty index; NA, not applicable.

Frailty was associated with a higher risk of SSI (OR = 4.24, 95% CI: 0.96-13.55, *P* = .026; Table 6). Interestingly, age was not a significant predictor of SSI. ASA class was the strongest predictor (ASA Class ≥III, OR = 10.83, 95% CI: 1.29-90.87, *P* = .018), followed by number of major comorbidities (number ≥2, OR = 10.49, 95% CI: 0.56-57.95, *P* = .028). Frailty was a stronger predictor than age, BMI, smoking, and mCCI score.

**Table 6.** Univariate Logistic Regression Analyses for Surgical-Site Infections by Strata

Risk index	Stratification	Surgical-site infection		
		*n*	Odds ratio (95% CI)	*P*-value
Age, years	<30 (Ref)	1326	NA	NA
	30-49	818	0.36 (0.05-1.40)	.190
	50-65	285	1.56 (0.34-5.26)	.509
	>65	49	3.05 (0.16-16.71)	.295
BMI, kg/m^2^	<24.9 (Ref)	1819	NA	NA
	25.0-29.9	446	3.53 (1.13-10.68)	.024
	>30	192	2.72 (0.40-11.37)	.213
No. of major comorbidities	0 (Ref)	2319	NA	NA
	1	138	4.66 (1.05-15.14)	.019
	≥2	21	10.49 (0.56-57.95)	.028
Smoking status	Nonsmoker (Ref)	2177	NA	NA
	Smoker	301	1.11 (0.17-4.06)	.888
ASA class	I—no disturb (Ref)	1301	NA	NA
	II—mild disturb	1055	6.84 (1.83-44.29)	.012
	≥III—severe disturb	116	10.83 (1.29-90.87)	.018
mFI-5 score	0 (Ref)	2338	NA	NA
	≥1	140	4.24 (0.96-13.55)	.026
mCCI score	0 (Ref)	2080	NA	NA
	1	250	2.51 (0.56-8.28)	.164
	≥2	148	2.84 (0.43-10.88)	.181

ASA, American Society of Anesthesiologists; mFI-5, modified 5-item frailty index; mCCI, modified Charlson comorbidity index; NA, not applicable.

Multivariate analysis was performed to account for other confounding variables. Frailty did not reach statistical significance, but multivariable logistic regression found frailty as the strongest independent predictor of 30-day aggregate complications (adjusted OR = 3.14, 95% CI: 0.86-12.36, *P* = .085; Table 7). ASA class was the strongest independent predictor for surgical-site complication (adjusted OR = 5.77, 95% CI: 01.45-38.37, *P* = .027). Notably, historic risk proxies, such as age, obesity, and smoking, were not significantly associated with complication risk.

**Table 7.** Multivariate Logistic Regression Analysis

Risk index	Overall 30-day complication			Surgical-site complication		
	Adjusted odds ratio	95% CI	Adjusted *P*-value	Adjusted odds ratio	95% CI	Adjusted *P*-value
Frailty	3.14	0.86-12.36	.085	1.73	0.24-14.21	.576
Age ≥ 50	1.58	0.72-3.27	.211	0.92	0.23-2.71	.895
BMI ≥ 30	1.13	0.59-2.05	.705	2.32	0.80-6.84	.119
ASA class > I	1.15	0.62-2.11	.658	5.77	1.45-38.37	.027
Smoking status	1.49	0.66-3.03	.304	0.81	0.80-6.84	.119
Charlson score	0.39	0.08-1.58	.211	1.11	0.10-9.40	.929

ASA, American Society of Anesthesiologists.

## DISCUSSION

Since the advent of breast implants in the 1960s,^22^ breast augmentation has become the top cosmetic procedure, according to the International Society of Aesthetic Plastic Surgery 2020 report.^23^ Recently, several studies have raised safety concerns with breast implants due to reports on their association with breast implant-associated anaplastic large-cell lymphoma and patient-reported breast implant illness.^24^ Although certain complication profiles have been investigated, an important question is which patients are most at risk and for what time period postoperation. One prospective study from the early 2000s reported that short-term complications following breast augmentation occurred most frequently within the first 3 months, but found these to be inconsequential to treatment management.^25^ As breast implant complication profiles continue to vary, consider patient risk profiles to evaluate their susceptibility. Being able to predict risk is especially important for breast implant patients, because these patients tend to be younger and have less comorbidities on average.

Previous studies in breast surgery have assessed the utility of the mFI-5 as a surgical-risk predictor in breast reconstruction,^17,26^ free-flap procedures,^27^ and mastectomies.^28^ Across all studies, increasing mFI-5 scores was significantly associated with postoperative complications. However, to this date and to our knowledge, no study has looked at frailty and breast augmentation.

In this retrospective study, mFI-5 frailty status was a consistently significant predictor of overall 30-day complications and SSIs. Our findings suggest that the mFI-5 was a superior predictor of breast augmentation complications compared to BMI, smoking status, diabetes, steroid use, and ASA classification. Although there was a low complication rate within our cohort, the large OR and effect size associated with mFI-5 prediction abilities highlight its significance as a clinically important tool.

In other words, our findings from the univariate and multivariate analyses show that frailty is associated with worse outcomes. While some *P*-values are just above the .05 cutoff, our results are likely still significant given the small sample size of frail patients, because most plastic surgeons do not perform breast augmentations on frail patients and frail patients tend to be older and are less likely to desire a breast augmentation, and the rare complication rate.

Our results demonstrate that age is a strong predictor of overall complications; patients >65 years of age have 5.86-fold increased odds of developing a complication. However, the majority of patients undergoing breast augmentation are young (mean 35.9 years old). Therefore, an understanding of other predictors that may affect surgical risk outside of age is crucial. Interestingly, age is not a component of the mFI-5. We demonstrate that comorbidities and mFI-5 (a measure of select comorbidities) have associations with an increased risk of adverse events. Interestingly, obesity was not found to be associated with overall complications. This difference from previous studies may be attributed to the fact that other studies looking at obesity and complications included breast-specific surgery outcomes, and obese patients in other studies frequently had other comorbidities.^29^ Our results may suggest that surgeons can safely operate on obese, elderly, and smoking patients as long as they do not have multiple comorbidities.

SSIs were one of the most common complications (0.44%) following breast augmentation. Frail patients were associated with more SSIs than nonfrail patients (*P* = .049). Likewise, through logistic regression analysis, mFI was shown to be a strong predictor of SSI, suggesting that frail patients have 4.24-fold increased odds of developing an SSI when compared with nonfrail patients (OR = 4.24, *P* = .026). More comorbidities, higher ASA class, and obesity all had increased associations with SSIs. Interestingly, age did show an increase in the odds of developing SSIs, but its impact was not significant. More accurately predicting SSI may prove to be increasingly important as SSI is a current proposed cause of capsular contracture.^30^ Capsular contracture, as stated earlier, is the most common complication following breast augmentation and frequently leads to reoperation.^5^

After controlling for other independent variables, the multivariate logistic regression model revealed mFI-5 was the strongest predictor of overall-day complications (OR = 3.14, 95% CI: 0.86-12.36, *P* = .085). Unfortunately, mFI-5 approached but did not reach statistical significance. Given the wide CI, this is likely in part due to the small number of complications and insufficient statistical power. In addition, due to the limited number of variables in the NSQIP database, the power of the effect of mFI-5 on complication outcomes might depend on the presence or absence of other factors, and future analysis with a larger patient population with more subgroup analysis may be able to determine its effect.

The NSQIP database is a large multi-institutional national study; however, the nature of the database leads to several limitations. NSQIP is designed to record surgical procedures across specialties and does not contain breast-specific complications, such as seroma, rippling, capsular contracture, malposition, and outcomes.^31^ Furthermore, NSQIP is limited to a 30-day window, and many complications, such as rippling, capsular contracture, double capsules, and late seromas, occur past this timeframe. This can lead to a false lower rate of complications. Likewise, our complication rate was 2.14%, consistent with other NSQIP studies looking at breast augmentations,^32^ but lower than single-institution breast augmentation complication studies.^2,3^ Because the NSQIP does not include data from office-based ORs, demographic and clinical characteristics may be subject to selection bias. This may be further compounded by any variations in reporting practices.

For future studies, it is critical to incorporate breast augmentation-specific outcomes. A larger sample of long-term data is also needed to better understand the relationship between surgical-risk indices and their associations with complication development. Increased information regarding complications will also lead to stronger associations between risk factors and outcomes, and can help us better understand how to surgically manage patients presenting for breast augmentation.

## CONCLUSIONS

Adequate preoperative risk stratification is crucial toward optimizing postoperative outcomes and patient safety. The present analysis demonstrates the clinical utility of using the mFI-5, a measure of frailty, with regards to the predictive ability for overall 30-day complications and SSIs in patients undergoing breast augmentation. Given the feasibility of the mFI-5, this tool offers surgeons a quick and robust assessment of frailty and predictive adverse outcomes within the preoperative evaluation process.
